# Psychological symptom patterns and vital exhaustion in outpatients with chronic obstructive pulmonary disease

**DOI:** 10.1186/1744-859X-10-32

**Published:** 2011-12-06

**Authors:** Athanasios Tselebis, Dionisios Bratis, Epaminondas Kosmas, Maria Harikiopoulou, Elpida Theodorakopoulou, Silvia Dumitru, Georgios Moussas, Athanasios Karkanias, Ioannis Ilias, Nikolaos Siafakas, Alexandros Vgontzas, Nikolaos Tzanakis

**Affiliations:** 1 Psychiatric Department, Sotiria General Hospital of Chest Diseases, Athens, Greece; 2 Pulmonary Rehabilitation Centre, Sotiria General Hospital of Chest Diseases, Athens, Greece; 3 Endocrine Department, Elena Venizelou Hospital, Athens, Greece; 4 Department of Thoracic Medicine, University of Crete, Medical School, Heraklion, Greece; 5 Psychiatric Department, University of Crete, Medical School, Heraklion, Greece; 6 Departement of Social Medicine, Laboratory of Epidemiology, University of Crete, Medical School, Heraklion, Greece

## Abstract

**Background:**

Several studies have reported high prevalence of anxiety and depression in chronic obstructive pulmonary disease (COPD) outpatients. Moreover, these patients share psychological or psychopathological characteristics that inhibit their ability to cope with the disease. In the present study we aimed to record the prevalence of psychological symptom patterns in a sample of Greek COPD outpatients and to assess which psychological factors (and to which degree) contribute to vital exhaustion (VE).

**Methods:**

The study included 139 COPD outpatients. We used the Symptom Checklist 90 - Revised (SCL-90-R) and the Maastricht Questionnaire (MQ) in order to evaluate psychological symptom patterns and VE, respectively.

**Results:**

The mean MQ score was 19.6, which is significantly higher than the corresponding score in the general population. Regarding the SCL-90-R dimensions, depression was the highest followed by somatization, obsessive-compulsive and anxiety dimensions. Additionally, a positive correlation was observed between the MQ and the SCL-90-R dimensions. MQ failed to demonstrate correlation with age, gender, education level or the severity of the disease. Depression seems to be responsible for 57.9% of the variation of VE, while obsessive-compulsiveness is responsible for an additional 2.4%. All the remaining dimensions of SCL-90-R had no statistically significant contributions.

**Conclusions:**

Our findings suggest the high prevalence of VE, together with high rates in most of the SCL-90-R dimensions with greater depression, somatization, obsessive-compulsiveness and anxiety in a Greek COPD group at various Global Initiative for Chronic Obstructive Lung Disease (GOLD) diagnostic criteria stages. The coexistence of such symptoms should be further assessed as an eventual unfavorable prognostic factor.

## Background

Chronic obstructive pulmonary disease (COPD) is characterized by chronic airflow limitation and a range of pathological changes in the lung, some significant extrapulmonary effects and important comorbidities that may contribute to the severity of the disease in individual patients [1]. The most common symptoms, but not specific for COPD, are breathlessness ('need for air'), excessive sputum production and chronic persistent cough [2].

Although there are several studies that have suggested that the prevalence of anxiety and depression is higher in patients with COPD than in patients with other chronic diseases, it is possible that there are certain psychological or psychopathological factors in these patients that influence their ability to cope with the disease [3].

Vital exhaustion (VE) is defined as a state characterized by unusual fatigue and lack of energy, increasing irritability and feelings of demoralization, typically attributed to prolonged psychological stress [4,5]. The concept of VE grew out of an interest in understanding the nature of the feeling of unusual tiredness that is reported among patients with cardiovascular diseases [6]. However, in a previous preliminary study we also found significantly high levels of vital exhaustion in patients with bronchial asthma [7].

It is known that mortality in COPD is more often due to cardiac rather than respiratory causes [8,9]. However, psychological comorbidities, and especially depressive symptoms, are independent prognostic factors for mortality among stable COPD patients [10].

Taking into account the limited information in the literature, especially in Greece, regarding the prevalence of overall psychological comorbidities and of VE in COPD outpatients, we aimed to record their prevalence and their associations in a sample of Greek COPD outpatients and to assess which psychological factors (and to which degree) contribute to VE.

## Methods

### Sample

We selected 139 COPD outpatients to participate in the study from the outpatients list of scheduled appointments in our hospital's clinics (among the largest respiratory diseases hospitals in Europe) by using an Excel random algorithm. No patient refused to participate in the study; the participants were approached at a referral center and were very motivated. We excluded patients who were aged > 80 years and patients diagnosed with major comorbidities such as heart failure, myocardial infarction, cerebrovascular disease, cancer, or severe orthopedic disorders as well as major mental disorders such as schizophrenia or mood disorder. Related information was obtained from the subjects' medical histories and medical records. Age, gender, family status and years of education were noted.

### Physiological measures

In order to determine COPD severity of our sample, a spirometric evaluation after 4 weeks without an acute exacerbation, before and after bronchodilation (200 μg salbutamol), was performed. We followed the Global Initiative for Chronic Obstructive Lung Disease (GOLD) diagnostic criteria, which classifies COPD severity (in relation to forced expiratory volume in 1 second (FEV_1_)/forced vital capacity (FVC) ratio (FEV_1_%) predicted) into four stages: stage I (mild COPD) = FEV_1 _> 80% predicted; stage II (moderate COPD) = FEV_1 _50% to 80% predicted; stage III (severe COPD) = FEV_1 _30% to 50% predicted; and stage IV (very severe COPD) = FEV_1 _< 30% predicted [1].

### Psychological measures

#### Symptom Checklist 90 - Revised (SCL-90-R)

The SCL-90-R is a 90-item self-report symptom inventory designed to reflect psychological symptom patterns of psychiatric and medical patients. Each item of the questionnaire is rated on a 5-point scale of distress from 0 (none) to 4 (extreme). The SCL-90-R consists of the following nine primary symptom dimensions: somatization (SOM, which reflects distress arising from bodily perceptions), obsessive-compulsive (OC, which reflects obsessive-compulsive symptoms), interpersonal sensitivity (IS, which reflects feelings of personal inadequacy and inferiority in comparison with others), depression (DEP, which reflects depressive symptoms, as well as lack of motivation), anxiety (ANX, which reflects anxiety symptoms and tension), hostility (HOS, which reflects symptoms of negative affect, aggression and irritability), phobic anxiety (PHO, which reflects symptoms of persistent fears as responses to specific conditions), paranoid ideation (PAR, which reflects symptoms of projective thinking, hostility, suspiciousness, fear of loss of autonomy), and psychoticism (PSY, which reflects a broad of symptoms from mild interpersonal alienation to dramatic evidence of psychosis) [11,12].

The SCL-90 takes between 12 and 20 min to complete. With regard to its reliability, the internal consistency coefficient α values for the nine symptom dimensions ranged from 0.77 for psychoticism, to a high of 0.90 for depression. Additionally, the few validity studies of the SCL-90-R demonstrate levels of concurrent, convergent, discriminant, and construct validity comparable to other self-report inventories. The SCL-90-R has been standardized and used in the Greek population and its reliability (Cronbach's α) for the total of the items is 0.97 [13,14]. The cutoff for the SCL-90-R subscales is 0.99 [14].

#### Maastricht Questionnaire (MQ)

The MQ is a self-report questionnaire consisting of 21 items that describe different aspects of VE, such as fatigue (for example, item 1: 'Do you often feel tired?'), lack of energy (for example, item 15: 'Do you sometimes feel that your body is like a battery that is losing its power?'), irritability (for example, item 12: 'Do little things irritate you more lately than they used to?'), demoralization (for example, item 13: 'Do you feel you want to give up trying?'), and depressive mood (for example, item 19: 'Do you feel like crying sometimes?') with a possible score range from 0 to 42 and good internal consistency (Cronbach's α, 0.89). The mean MQ score in a healthy population is 8.8 (± 8.7), with a cutoff > 18 [15,16]. The questionnaire has been adapted into the Greek language and its reliability is considered to be satisfactory (Cronbach's α, 0.87) [16].

Regarding sample size calculation, for 16 independent variables, and assuming an α level of 0.05, a medium anticipated effect size (f2) of 0.15 and a desired statistical power level of 0.8, Cohen's formula [17] gives a required sample size of 142. Our sample size (see next section) just falls short of this level, but is amply sufficient for testing individual predictors using the method of Green [18], which, for 16 independent variables, gives a sample size of 119.

Statistical analyses were performed using the Student's t test, Pearson's correlation and stepwise (backward eliminating) multiple linear regression (that is, removing one parameter at a time with the highest *P *value). Statistical significance was set at *P *< 0.05.

The hospital ethics committee approved the study and all participants provided their consent. No financial support was necessary.

## Results

In all, 139 COPD outpatients were included in the study: 107 men (mean age = 64.92, SD = 8.16) and 32 women (mean age = 63.66, SD = 7.74), with mean duration (in years) of education 10.9 (SD = 4.07). The mean FEV_1 _was 43.5 ± 14.6% predicted (Table 1). There was no statistical difference between males and females, regarding age, level of education, and FEV1% predicted (t test *P *> 0.05).

**Table 1:** Demographics and baseline characteristics

	Male (N = 107)		Female (N = 32)		Total (N = 139)		t Test (df = 137)
		
	Mean	SD	Mean		Mean	SD	
Age	64.91	8.15	63.65	7.74	64.62	8.05	*P *> 0.05
Education (years)	10.59	4.05	11.9	4.027	10.89	4.07	*P *> 0.05
FEV_1_% of predicted	41.45	20.23	52.17	23.26	43.48	21.15	*P *= 0.05

Severity as per Global Initiative for Chronic Obstructive Lung Disease (GOLD) diagnostic criteria: mild/moderate/severe/very severe 10/19/47/35.

FEV_1_% = forced expiratory volume in 1 second (FEV_1_)/forced vital capacity (FVC) ratio.

With regard to gender, females presented with higher mean scores in depression, obsessive-compulsiveness, anxiety, hostility and somatization than males (t test *P *< 0.05, Table 2). The mean MQ score was 19.6 (Table 2), which is significant higher than the corresponding to the general population (8.7) adjusted for age and sex [16] (sample t test *P *< 0.05). However, gender was not associated with vital exhaustion (t test *P *> 0.05).

**Table 2:** Mean, SD, median for Symptom Checklist 90 Revised (SCL-90-R) and the Maastricht Questionnaire (MQ) with regard to gender

	Male (N = 107)			Female (N = 32)			Total (N = 139)			t Test (df = 137)
		
	Mean	SD	Median		SD	Median	Mean	SD	Median	
SOM	0.72	0.46	0.58	1.04	0.69	0.83	0.80	0.54	0.66	*P *< 0.01
OC	0.68	0.50	0.50	1.08	0.66	0.90	0.77	0.56	0.70	*P *< 0.001
IS	0.41	0.47	0.22	0.63	0.56	0.49	0.46	0.50	0.33	*P *> 0.05
DEP	0.80	0.60	0.61	1.19	0.64	1.07	0.89	0.63	0.69	*P *< 0.01
ANX	0.60	0.53	0.50	0.91	0.71	0.70	0.67	0.59	0.50	*P *< 0.01
HOS	0.47	0.63	0.33	0.80	0.74	0.66	0.54	0.67	0.33	*P *< 0.05
PHO	0.36	0.58	0.14	0.45	0.72	0.28	0.38	0.61	0.14	*P *> 0.05
PAR	0.43	0.55	0.33	0.56	0.68	0.33	0.46	0.58	0.33	*P *> 0.05
PSY	0.15	0.34	0.00	0.30	0.44	0.20	0.18	0.37	0.00	*P *> 0.05
MQ	18.96	8.56	18.00	21.84	9.54	21.00	19.62	8.84	19.00	*P *> 0.05

ANX = anxiety; DEP = depression; IS = interpersonal sensitivity; HOS = hostility; OC = obsessive-compulsive; PAR = paranoid ideation; PHO = phobic anxiety; PSY = psychotism; SOM = somatization.

Regarding the SCL-90-R dimensions, depression presented the higher mean score, followed by somatization, obsessive-compulsive and anxiety dimensions (Table 2).

One out of two patients presented a clinically significant elevation (score > 0.99) in at least one dimension of the SCL-90-R. A total of 36% of the participants presented with depression, while 33% presented somatization, 30.9% obsessive-compulsiveness and 23.7% anxiety (Table 3).

**Table 3:** Patients (N, %) with clinically significant symptoms on the Symptom Checklist 90 Revised (SCL-90-R) and the Maastricht Questionnaire (MQ)

Scale	Factor	N	%
SCL-90-R	Without psychopathology > 0.99	62	44.6
	
	Somatization (SOM) > 0.99	47	33.8
	
	Obsessive-compulsive (OC) > 0.99	43	30.9
	
	Interpersonal sensitivity (IS) > 0.99	19	13.7
	
	Depression (DEP) > 0.99	50	36.0
	
	Anxiety (ANX) > 0.99	33	23.7
	
	Hostility (HOS) > 0.99	28	20.1
	
	Phobic anxiety (PHO) > 0.99	18	12.9
	
	Paranoid ideation (PAR) > 0.99	23	16.5
	
	Psychoticism (PSY) > 0.99	7	5.0

MQ	Vital exhaustion > 18	71	51.1

We observed a positive correlation between depression and all the other dimensions of SCL-90-R (Pearson correlation *P *< 0.01), while the stronger correlation was observed with obsessive-compulsiveness (Pearson correlation r = 0.700, *P *< 0.01) and anxiety (Pearson correlation r = 0.700, *P *< 0.01). No correlation was found between SCL-90-R dimensions and age or education level.

Additionally, a positive correlation was observed between MQ and SCL-90-R dimensions (Pearson correlation *P *< 0.01, Table 4). MQ failed to demonstrate correlation with age, education level or FEV1% of predicted (Pearson correlation *P *> 0.05).

**Table 4:** Correlations between the Symptom Checklist 90 Revised (SCL-90-R) and the Maastricht Questionnaire (MQ)

	MQ	SOM	OC	IS	DEP	ANX	HOS	PHO	PAR
SOM	0.587*								

OC	0.624*	0.601*							

IS	0.404*	0.564*	0.628*						

DEP	0.756*	0.683*	0.665*	0.608*					

ANX	0.600*	0.719*	0.612*	0.620*	0.780*				

HOS	0.417*	0.517*	0.565*	0.663*	0.570*	0.595*			

PHO	0.440*	0.445*	0.493*	0.443*	0.600*	0.697*	0.303*		

PAR	0.331*	0.539	0.587*	0.770*	0.495*	0.523*	0.635*	0.392*	

PSY	0.381*	0.545	0.618*	0.727*	0.580*	0.657*	0.487*	0.565*	0.694*

*P < 0.01.

ANX = anxiety; DEP = depression; IS = interpersonal sensitivity; HOS = hostility; OC = obsessive-compulsive; PAR = paranoid ideation; PHO = phobic anxiety; PSY = psychotism; SOM = somatization.

Finally, FEV_1_% of predicted presented a weak correlation only with somatization (Pearson correlation: r = 0.267, *P *< 0.05), while there was no correlation with MQ (Pearson correlation: r = 0.02, *P *> 0.05).

In order to examine the factors that participate to the elevation of MQ score we used stepwise multiple regression. We included all nine symptom dimensions of the SCL-90-R: depression seems to be responsible for 57.9% of the variation of vital exhaustion (F_1,137 _= 186.77, *P *< 0.001), while obsessive-compulsiveness is responsible for an additional 2.4% (F_1,136 _= 8.14, *P *< 0.001). All the remaining dimensions of SCL-90-R had no statistically significant contributions.

## Discussion

The main findings of our research suggest the high prevalence of VE together with high rates in most of the SCL-90-R dimensions with greater depression, somatization, obsessive-compulsiveness and anxiety. In a previous study using the SCL-90-R, the authors found similar rates in somatization, depression, anxiety and hostility [3]. These observations, in agreement with other findings in the literature, underline the association of psychological status of Greek COPD patients with their ability to cope with the disease and subsequently with their quality of their life [19,20].

It is known that psychological characteristics interact with physical symptoms and play an important role in how COPD patients experience and manage their disease [21]. According to this finding, the investigation of the prevalence of the psychological symptoms of these patients is established as necessary.

However, the relevant psychological status studies have mainly been focused on the prevalence of anxiety and depression, which often appear together in these patients [22-26]. The prevalence of depression among outpatients with COPD is substantially greater than lifetime rates in the general population (ranging between 10% and 42% in the former, compared to approximately 5% in the latter). Correspondingly, the prevalence of anxiety varies from 10% to 19% [26], a percentage that is higher than the 15% reported in the general population [27,28]. Higher predominance of depression and anxiety was observed in patients with COPD compared to patients that suffered from other chronic respiratory disorders, such as bronchial asthma and tuberculosis [28]. Both depression and anxiety are significantly associated with decreased functional status and worse health status when compared to those of patients without psychological symptoms, even after controlling for the effects of overall health status [24,29,30]. In a previous study we showed the high prevalence of anxiety and depressive symptoms, together with their association with alexithymia ('lack of words for emotions') in Greek COPD outpatients [31].

In another relevant previous study [32], which used the SCL-90-R for the evaluation of psychological symptoms in COPD patients, the authors concluded that somatization, anxiety and depression correlated with behavioral impairment of multiple daily activities.

Regarding VE, there are no studies in the literature for COPD patients. However, in a study concerning fatigue (which is the main symptom of VE) it was shown that almost all participants with COPD experienced this symptom. Furthermore, fatigue affected greater proportions of participants than either anxiety or depression [33], a finding similar to our results.

However, depression and vital exhaustion have important symptoms in common, most notably increased fatigue and irritability, which raises the question as to what extent these constructs may differ from each other [34]. Two previous studies that were designed to investigate this question concluded that VE is characterized by loss of vigor and fatigue rather than depressed mood [35]. Furthermore, VE is related more to loss of energy and bodily symptoms, whereas depression is more related to dysfunction attitudes, lack of purpose, low self-efficacy and hostility [36].

In a previous study in patients following first myocardial infarction, depression and fatigue were highly correlated and their association was not attributable to comorbid physical illnesses or the tendency of the MQ to measure depression [37]. However, according to the findings of the present study, depressive symptoms have an effect on the degree of VE, leading to a more complex clinical aspect; their prognostic value needs to be assessed with further studies.

COPD contributes to an impaired quality of life [38]. Fatigue, which is the main feature of VE, imposes limitations on motivation, concentration and the ability to engage in everyday activities, engendering frustration and a sense of loss of control [33]. Finally, COPD severity, as determined by FEV_1_% of predicted, failed to demonstrate correlation with VE and SCL-90-R dimensions, with the exception of somatization (which may reflect the underlying symptoms of the disease). This observation is in line with the hypothesis that FEV1% of predicted does not mediate all the aspects of the disease and demonstrates poor correlation with quality of life [39]. It is possible that the patients construe disease seriousness with a subjective view, which contributes to the development of the levels of psychological distress [31].

This study has limitations: the most important relates to the fact that the design did not include verification of the hypothesis that the simultaneous presence of a high degree of psychological comorbidity and VE is possibly a predictor of adverse outcomes in COPD. This hypothesis should be tested with different types of further studies.

## Conclusions

Our findings suggest the high prevalence of VE together with high rates in most of the SCL-90-R dimensions with greater depression, somatization, obsessive-compulsiveness and anxiety in a Greek COPD group at various GOLD stages. The coexistence of such symptoms should be further assessed as an eventual unfavorable prognostic factor.

## Competing interests

The authors declare that they have no competing interests.

## Authors' contributions

AT conceived the experiment, designed the study, performed the psychological measures, collected data, carried out the statistical analysis and drafted the paper; DB performed the psychological measures, carried out the statistical analysis and drafted the paper; EK performed the physical measures and helped draft the paper; MH, ET and SD performed the physical measures; GM and AK helped draft the paper; II carried out the statistical analysis and helped draft the paper; NS and AV supervised the study; NT carried out the statistical analysis, helped draft the paper and supervised the study. All authors read and approved the final manuscript.
